# Ohmic contact between iridium film and hydrogen-terminated single crystal diamond

**DOI:** 10.1038/s41598-017-09380-1

**Published:** 2017-09-22

**Authors:** Yan-Feng Wang, Xiaohui Chang, Shuoye Li, Dan Zhao, Guoqing Shao, Tianfei Zhu, Jiao Fu, Pengfei Zhang, Xudong Chen, Fengnan Li, Zongchen Liu, Shuwei Fan, Renan Bu, Feng Wen, Jingwen Zhang, Wei Wang, Hong-Xing Wang

**Affiliations:** Institute of Wide Band Gap Semiconductors, School of Electronics and Information Engineering, Xi’an Jiaotong University, Xi’an, &10049 PR China

## Abstract

Investigation of ohmic contact between iridium (Ir) film and hydrogen-terminated single crystal diamond has been carried out with annealing temperature from 300 to 600 °C in argon (Ar) and hydrogen ambient. Electrodes were deposited on hydrogen-terminated single crystal diamond by electron beam evaporation technique, and specific contact resistivity has been measured by transmission line model. The interface between Ir film and hydrogen-terminated single crystal diamond was characterized by transmission electron microscopy and energy dispersive X-ray spectroscopy. Theoretical calculation value of barrier height between Ir film and hydrogen-terminated single crystal diamond was around −1.1 eV. All results indicate that an excellent ohmic contact could be formed between Ir film and hydrogen-terminated single diamond.

## Introduction

Diamond exhibits many outstanding properties such as good light transmittance, effective resistance to radiation damage, large bandgap, high breakdown voltage, high thermal conductivity, high carrier mobilities etc., having potential applications in the fields of wide range optical transparent window material, coating tools, especially in the field of electron devices which can work in high frequency, high power, high temperature as well as corrosive environment^1–15^. However, the activation energies of boron and phosphorus dopants are about 370 meV and 570 meV, which are too large to be activated at room temperature^16^. A two dimensional hole gas channel which several nanometers below diamond surface indicate that diamond sample hydrogen termination (H-termination) bonds are produced by treating with hydrogen plasma, resulting in a p-type conduction layer and promoting the development of diamond-based devices. This p-type conduction layer has a sheet carrier density and mobility of 10^13^ cm^−2^ and 50–150 cm^2^V^−1^s^−1^, respectively, nearly temperature-independent between 20 K and 300 K^17,18^. To enhance better performance of diamond electronic devices, an excellent ohmic contact between electrode metal layer and diamond is needed^9,19–24^. Up to now, many investigations of ohmic contact between metal and diamond have been reported, such as gold (Au), platinum (Pt) and palladium (Pd) on hydrogen-terminated single crystal diamond, all of which yield important role in diamond device development so far^9,25,26^. However, Au could peel off during fabrication process, which indicates quite poor adherence of Au on hydrogen-terminated single crystal diamond film^9^. While Pt and Pd could be corroded in harsh environment, which can not adapt to the application of diamond electronic devices^27^.

Iridium (Ir) as another one excellent metal overcomes the shortcomings mentioned above. The properties of most corrosion resistant, large work function (5.27 eV), high melting point, high electrical conductivity make Ir a promising candidate form ohmic contact with hydrogen-terminated single crystal diamond^28,29^. To authors’ knowledge, few investigations on Ir ohmic contact with hydrogen-terminated single crystal diamond were reported. In this work, properties of Ir on hydrogen-terminated single crystal diamond have been investigated by transmission line model (TLM), scanning electron microscopy (SEM), X-ray photoelectron spectroscopy (XPS) and transmission electron microscopy (TEM) techniques.

## Methods

**Figure 1:**
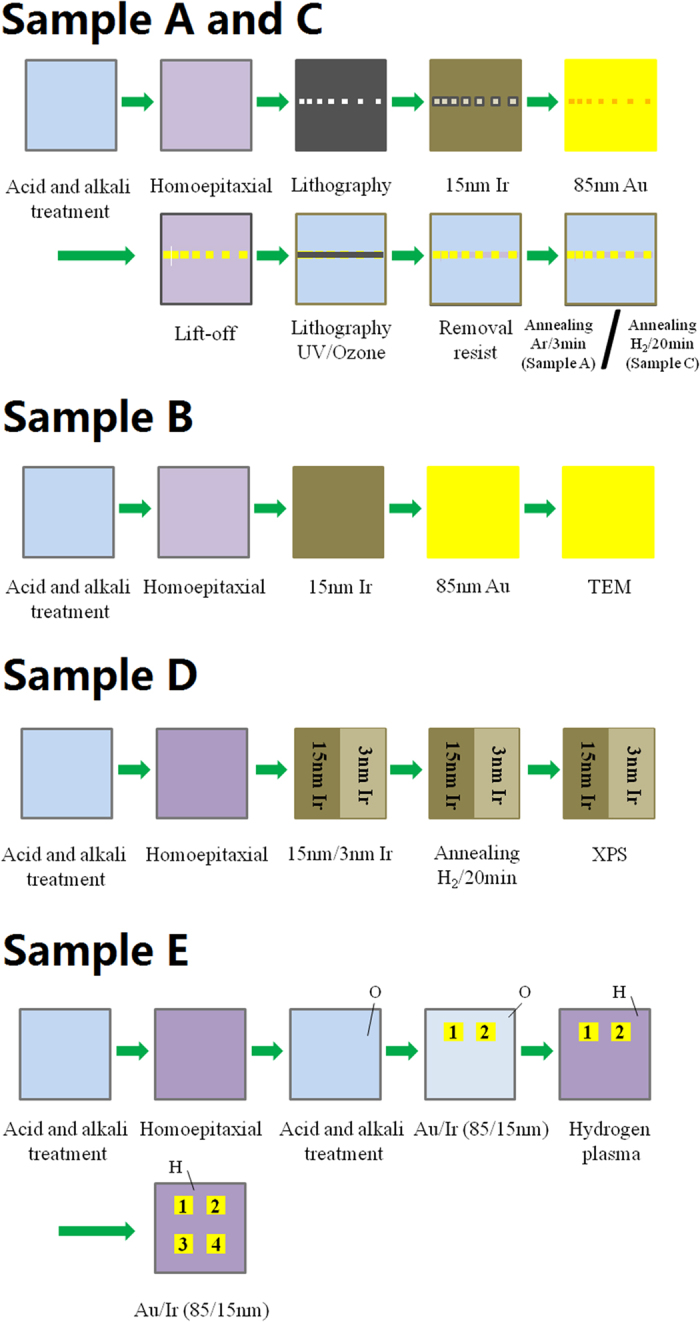
Schematic of fabricating process (**a**) for sample A (**b**) and sample B.

The schematic of detailed fabricating process is shown in Fig. 1. Five CVD synthesized (001) IIa single crystal diamonds (Elemetsix Corp.) substrates, labeled the sample A, B, C, D and E in dimension of 3 × 3 × 0.5 mm^3^ respectively, were treated in an acid mixture of H_2_SO_4_:HNO_3_:HClO_4_ = 31.2:36:11.4 at 250 °C for 1 hour and then in a mixed alkali of NH_4_OH:H_2_O_2_:H_2_O = 4:3:9 at 80 °C for 10 minute to remove non-diamond phase. The 200 nm undoped homoepitaxial single crystal diamond films with H-termination were grown on five substrates, respectively. The growth of signal crystal diamond films was carried out in commercial microwave plasma CVS system (AX5200 Seki Technotron Corp.) and the detailed epitaxial growth process and conditions have been reported in our previous work^9^. The photolithographic technique was used to pattern TLM configuration with 100 × 100 μm^2^ area and spaced from 5 to 30 μm on sample A and C. After electrodes done, 15 nm Ir and 85 nm Au were deposited on sample A, B and C by electron beam evaporation technique at room temperature. For sample A and C, patterned photoresist was used to protect Au/Ir electrodes and conductive channels under UV/ozone for isolation. Finally, ohmic contact was investigated by annealing sample at several temperatures from 300 to 600 °C in Ar ambient with 760 Torr pressure for 3 minutes and in 35 Torr pressure hydrogen ambient for 20 minutes for sample A and C, respectively. Cross-section of sample B was characterized by TEM. To fabricate 15 nm Ir on half of diamond and 3 nm Ir on the other half of diamond, for sample D, photolithographic and EB technique were used for a grown undoped homoepitaxial single crystal diamond film. Then sample D was annealed at 600 °C for 20 minutes in hydrogen ambient Sample E, after 200 nm undoped homoepitaxial single crystal diamond film was grown, was treated by acid mixture (H_2_SO_4_:HNO_3_ = 1:1) at 250 °C for 2 h to form oxygen-termination (O-termination) on surface. After that, two electrodes (electrode 1 and 2) with the size of 100 × 100 μm^2^ and spaced 10 μm Au/Ir (85/15 nm) were fabricated using photolithography and electron beam evaporation techniques. Then, sample E was treated by a hydrogen plasma to form H-termination on diamond surface. Next, electrodes 3 and 4 with the size of 100 × 100 μm^2^ and spaced 10 μm Au/Ir (85/15 nm) were fabricated using photolithography and electron beam evaporation techniques. After the sample E was prepared, I-V properties of electrodes 1, 2 and 3, 4 were measured. Thereafter, sample E was annealed at hydrogen ambient and 600 °C for 20 min. Furthermore, I-V properties of electrodes 1, 2 and 3, 4 were measured.

## Results and Discussion

After the growth of undoped homoepitaxial single crystal diamond on sample A, the surface morphology was evaluated by atomic force microscopy. The sample has a smooth surface, roughness about 0.345 nm, which is important for ohmic contact between Ir film and hydrogen-terminated single crystal diamond. Hall measurement of undoped homoepitaxial single crystal diamond was carried out with four tungsten probes pinned on four equidistant Pd circular electrodes at room temperature and atmosphere pressure, by which the carrier density, square resistance and mobility were evaluated and got the results of 2.63 × 10^13^ cm^−2^, 5.1 kΩ, 46 cm^2^V^−1^s^−1^, respectively.

**Figure 2:**
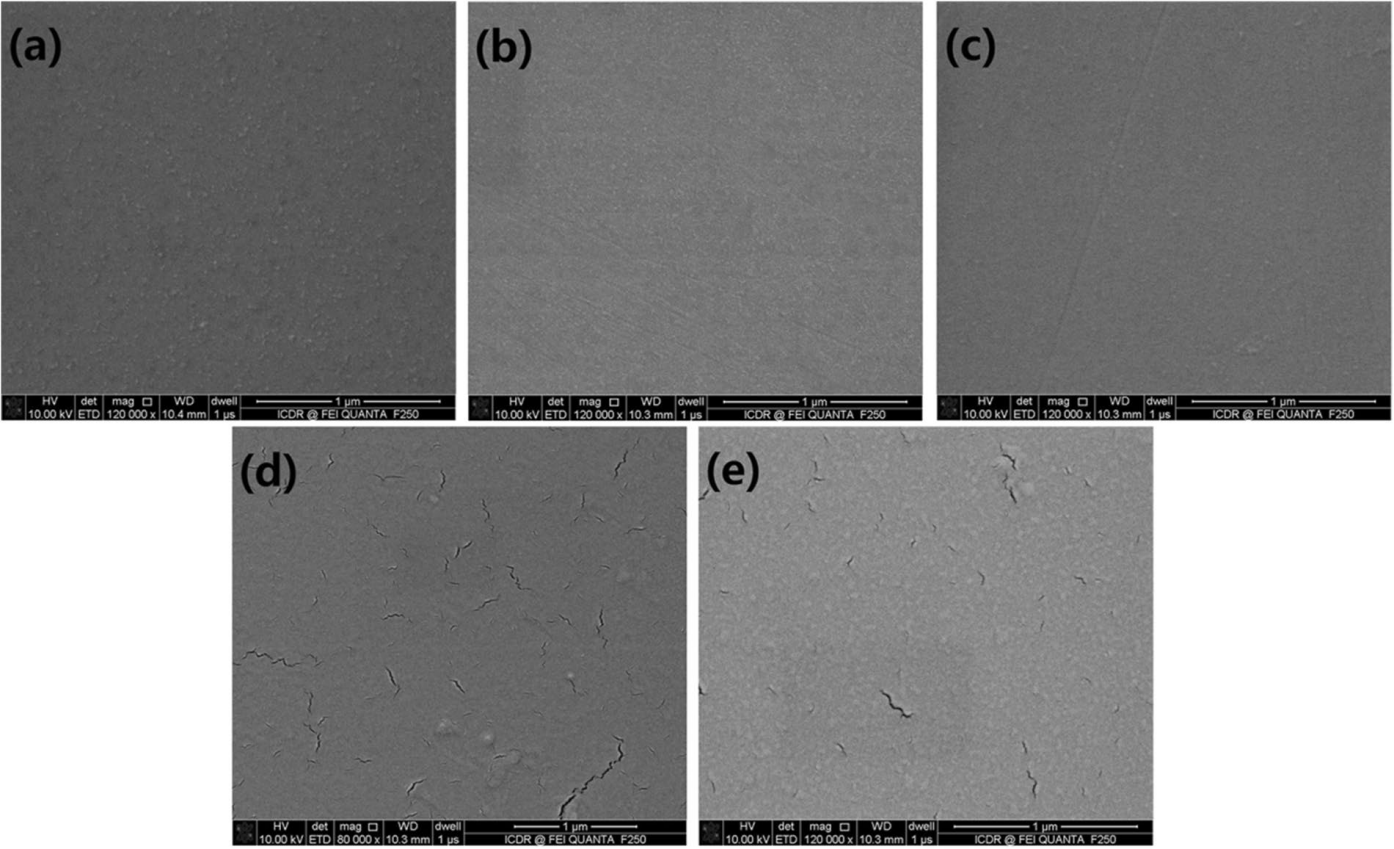
SEM images of Ir films on hydrogen-terminated single crystal diamond surface. (**a**) Thickness of Ir is 5 nm. (**b**) Thickness of Ir is 10 nm. (**c**) Thickness of Ir is 15 nm. (**d**) Thickness of Ir is 20 nm. (**e**) Thickness of Ir is 30 nm.

SEM images of Ir films on diamond surface with variable thicknesses from 5 to 30 nm are shown in Fig. 2. When the thicknesses were less than 15 nm (Fig. 2a–c), Ir films were continuity; subsequent the thickness more than 20 nm (Fig. 2(d and e)) the cracks appear on the surface of as-deposited Ir films. Considering the models of Ir brittle fracture at temperatures below 1000 °C, these cracks on Ir surface are intrinsic, which could be ascribed to the very strong and directed atomic binding forces^30,31^. Therefore, a 15 nm Ir film was deposited on the hydrogen-terminated single crystal diamond, and then a 85 nm Au film, no intermediate product with Ir, was deposited on Ir film to increase the thickness of electrodes

**Figure 3:**
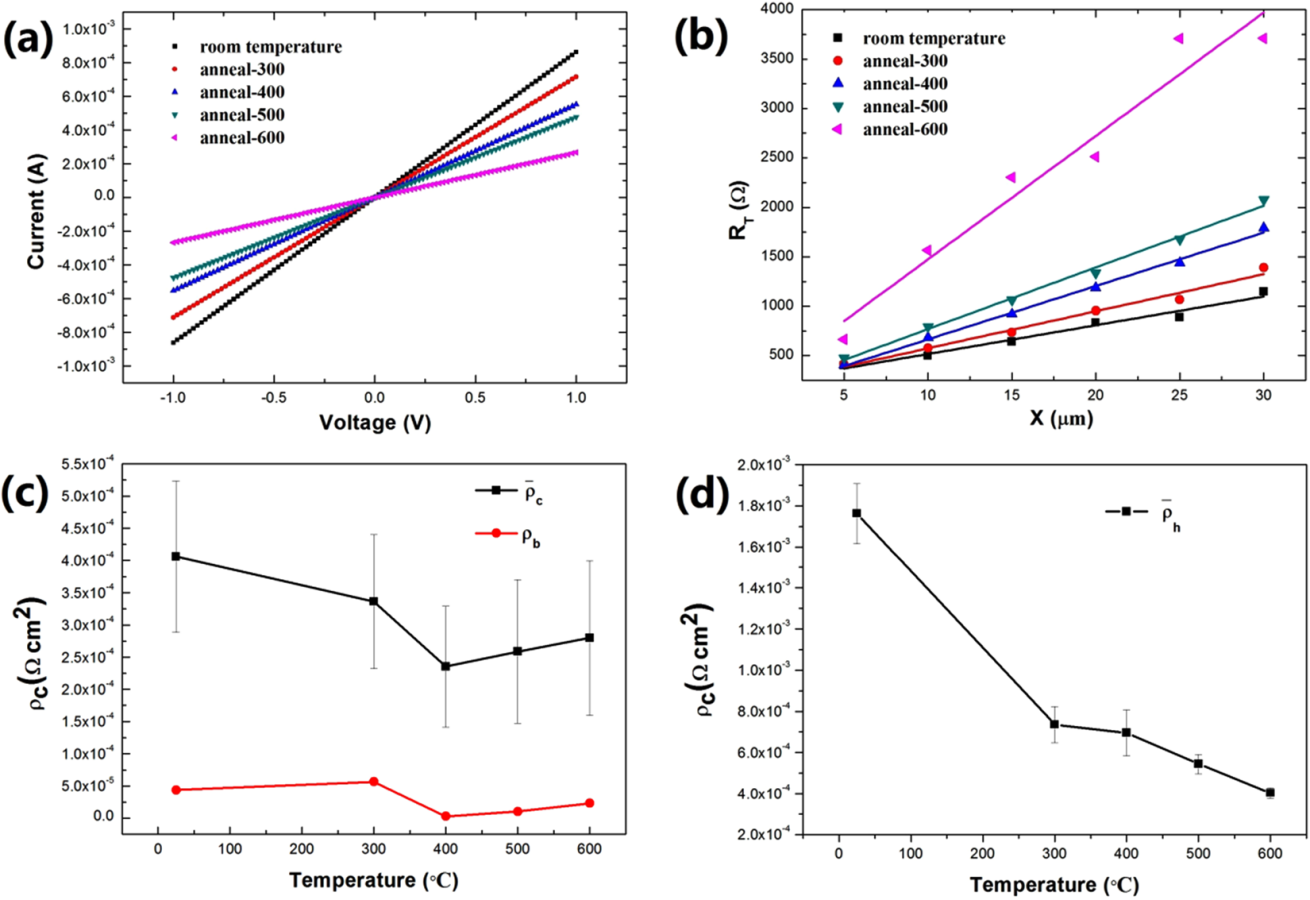
Results of electrical measurement for sample A and C annealed at different temperatures in Ar and hydrogen ambient. (**a**) I-V properties of Au/Ir electrodes on sample A annealed at several temperatures up to 600 °C. (**b**) Linear fitting curves of the same TLM pattern at different annealing temperature of sample A. (**c**) Specific contact resistivity values of sample A. Black line means average specific contact resistivity. Red line means one of TLM configurations’ specific contact resistivity values. (**d**) Average specific contact resistivity values of sample C.

The results of electrical measurement for sample A and C are shown in Fig. 3. I-V properties measured at two same Au/Ir electrodes on sample A and annealed at several temperatures up to 600 °C for 3 minutes in Ar ambient (Fig. 3(a)) have illustrated that an ohmic contact formed between Au/Ir electrodes and hydrogen-terminated single crystal diamond. As the annealing temperature increases, the linear slope decreases shown in Fig. 3(a), indicating the increase of resistance between the measured Au/Ir electrodes, which could be attributed to the reduction of absorption and H-termination bonds on the diamond surface during annealing process^32,33^. Linear fitting curves of the same TLM pattern at different annealing temperatures of sample A shown in Fig. 3(b). The results obtained at one annealing temperature are almost on the same line, which suggests a good behavior of ohmic contacts between Ir film and hydrogen-terminated single crystal diamond. Average specific contact resistivity (${\overline{{\rm{\rho }}}}_{{\rm{c}}}$) values of sample A were obtained by measuring 6 TLM with the same configurations at different annealing temperatures as shown in Fig. 3(c). The minimum of ${\overline{{\rm{\rho }}}}_{{\rm{c}}}$ value is 2.3 × 10^−4^ Ω cm^2^ at 400 °C. The ${\overline{{\rm{\rho }}}}_{{\rm{c}}}$ values decrease with increasing of annealing temperature below 400 °C, and that increase with increasing of annealing temperature above 400 °C. The decreasing tendency of ${\overline{{\rm{\rho }}}}_{{\rm{c}}}$ value lead to the improvement of ohmic contact in sample A, which could be ascribed to the interface state change between Ir and hydrogen-terminated diamond at annealing process. The ${\overline{{\rm{\rho }}}}_{{\rm{c}}}$ value with increasing tendency is possibly attributed by reduction of absorption and H-termination bonds during annealing process. One of TLM configurations’ specific contact resistivity values of sample A are shown in Fig. 3(c) as ρ_b_ (red line), and the best ρ_b_ is 2.9 × 10^−6^ Ω cm^2^ at 400 °C. To investigate the effect of specific contact resistivity with annealing time, sample C annealing time was extended to 20 minutes. However, when hydrogen-terminated diamond is annealed at high temperature for longer time, the thermal desorption of H-termination bonds between Au/Ir electrodes would happen on the diamond surface, and the resistance properties between Au/Ir electrodes becomes too lager to be evaluated. Fortunately, hydrogen atoms can passivate dangling bonds during annealing at hydrogen ambient^34^. Therefore, in order to reduce the thermal desorption of H-termination bonds, sample C was annealed in hydrogen ambient. The average specific contact resistivity (${\overline{{\rm{\rho }}}}_{{\rm{h}}}$) values (Fig. 3(d)) of sample C annealed from 300 °C to 600 °C, whose measurement method is the same as that used for sample A. The results show that the ${\overline{{\rm{\rho }}}}_{{\rm{h}}}$ in hydrogen ambient decreases with the annealing temperature increasing, which is different from that in Ar ambient. The reasons of this difference will be investigated and discussed in our future work.

We are aware of some references in which ρ_c_ value has been achieved and are vary from ours result 2.9 × 10^−6^ Ω cm^2^. For instance, Hou and co-workers reported the ρ_c_ value of Au/diamond is 5.43 × 10^−4^ Ω cm^2^ in ref.^20^. and Ye *et al*. found the value is 1 × 10^−6^ Ω cm^2^ in ref.^35^. The ρ_c_ value of Al/Si/diamond reported is 2 × 10^−2^ Ω cm^2^ and 2.3 × 10^−7^ Ω cm^2^ by Werner *et al*. in ref.^23^. The ρ_c_ value of Ti/diamond reported by Werner, *et al*. is 10^−4^ Ω cm^2^ in ref.^22^ and is 10^−7^ Ω cm^2^ found by Hiroshi and co-workers in ref.^36^. Au/Ti/diamond annealed at 700 °C for 20 minutes in hydrogen ambient studied by our laboratory ρ_c_ value is 9.8 × 10^−4^ Ω cm^2^. Many reasons can lead and attribute to these variable results fluctuations such as diamond quality, annealing conditions, doping concentration, carbide etc. All in all, Ir film has potential to perform excellent ohmic contact with hydrogen-terminated single crystal diamond.

**Figure 4:**
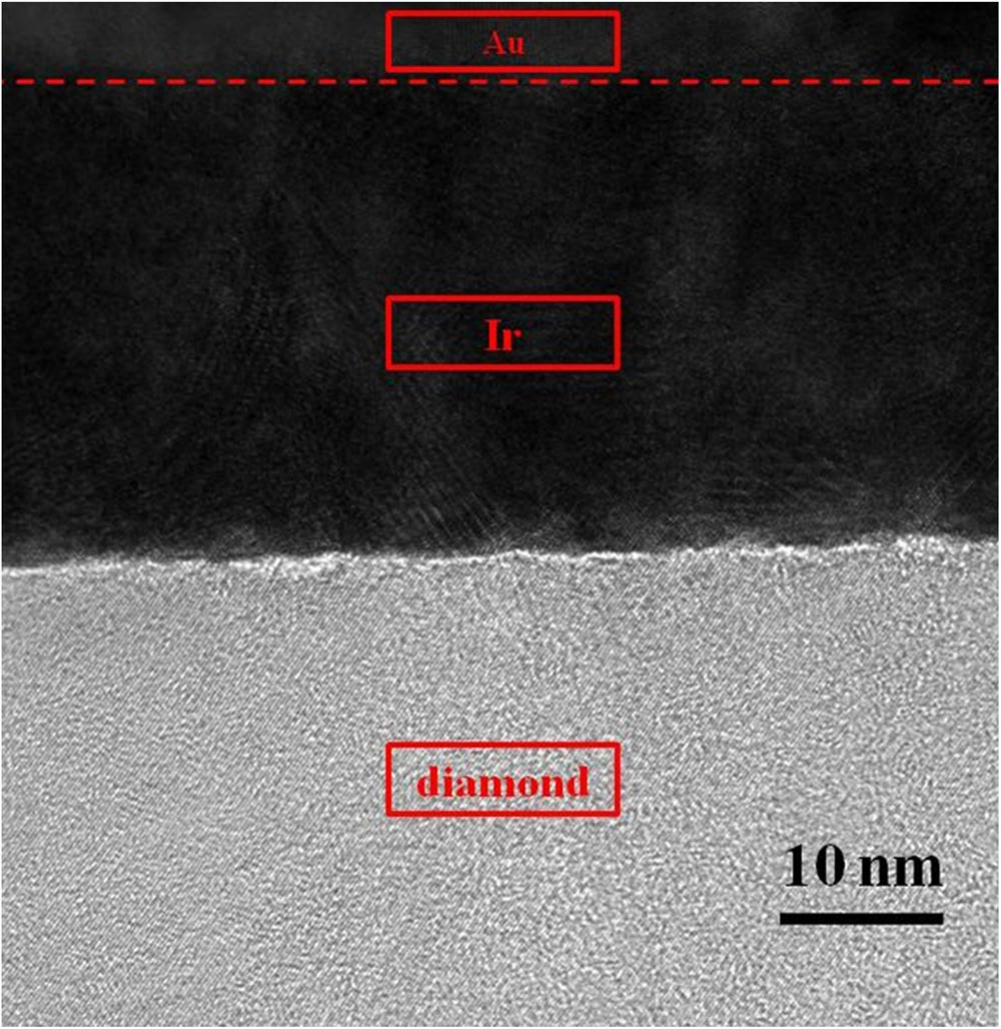
Image of high-resolution TEM for cross-section between Ir and hydrogen-terminated single crystal diamond in sample B.

**Figure 5:**
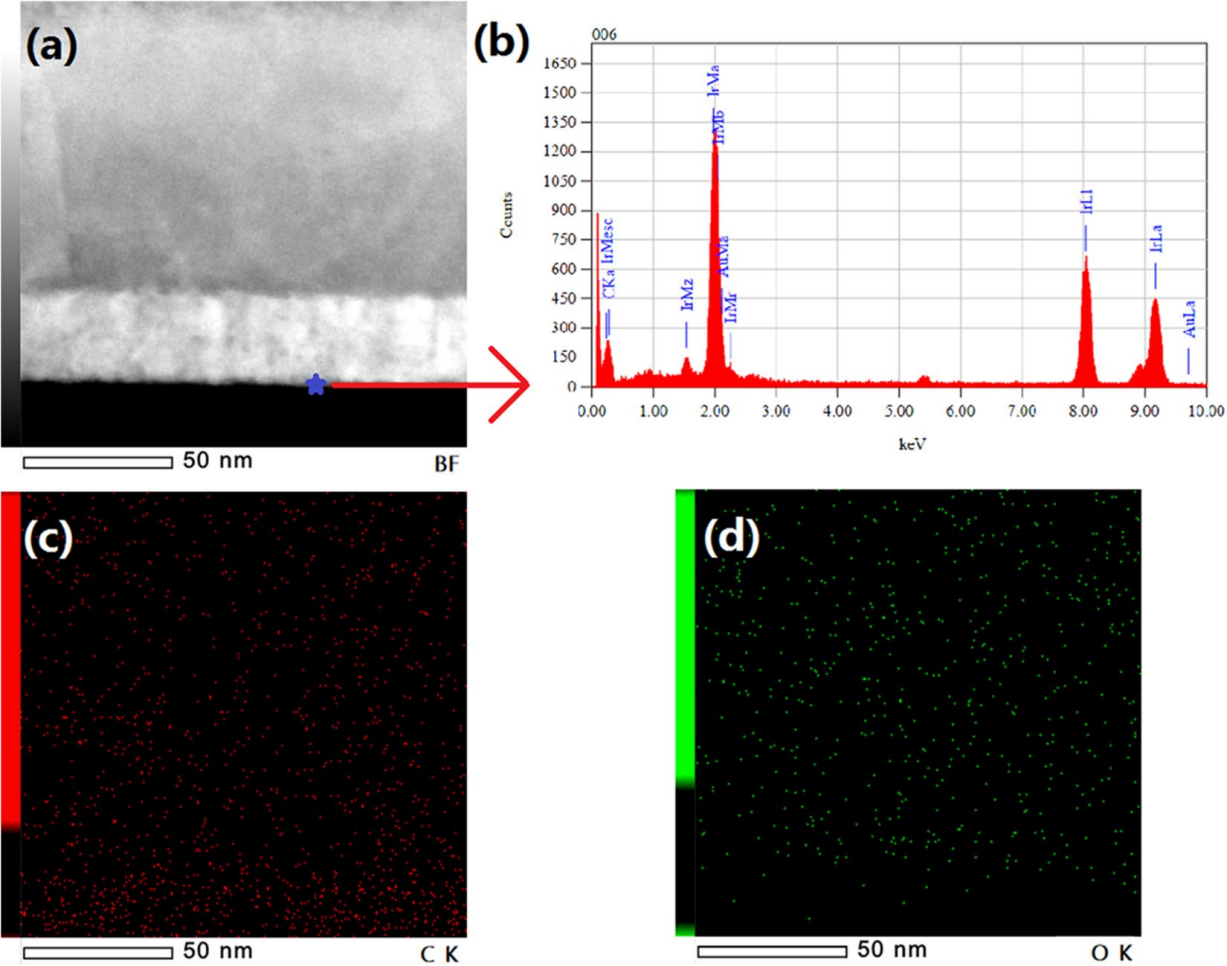
(**a**) Cross-section TEM image and the EDX measurement point of sample B (**b**) EDX result of marked point. (**c**) EDX elemental mapping of C (**d**) EDX elemental mapping of O.

In order to investigate the reason of forming ohmic contact between Ir film and hydrogen-terminated single crystal diamond, TEM technique was used to show interface between Ir and diamond. Tilt cross-section was prepared in sample B by focused ion beam technique to increase the observation range. Figure 4 shows image of high-resolution TEM for cross-section between Ir and diamond in sample B. In Ir layer, there are different lattice directions appeared, suggesting that Ir film is polycrystalline. It’s worth noting that buffer layer seems not exist between Ir film and hydrogen-terminated single crystal diamond. In addition, interface between Ir and diamond is almost flat, which indicates that no strong chemical reaction occurred during deposition process. In order to investigate whether intermediate product is formed between Ir film and hydrogen-terminated single crystal diamond during deposition process, energy dispersive X-ray spectroscopy (EDX) is used for elemental analysis. Figure 5(a) shows the cross-section TEM image and the EDX measurement point of sample B. Figure 5(b) exhibits the measurement result, indicating that only Ir and carbon (C) exist in this area without oxidized. The EDX elemental mapping of C and oxygen (O) in cross-section of sample B was also carried out. C and O distributions are uniform in the area of Ir and interface between Ir and diamond, indicating Ir is not carbonized and oxidized during deposition process, as shown in Fig. 5(c) and (d). These measurement results are identical with theory that Ir has tolerant of harsh condition without carbonization or oxidation. Therefore, no intermediate product is formed at interface between Ir film and hydrogen-terminated single crystal diamond, which could be one reason of forming ohmic contact.

Another reason of forming ohmic contact between Ir and hydrogen-terminated single crystal diamond could be ascribed to the large work function of Ir (5.27 eV). Barrier height (Φ_B_) is an important parameter to evaluate contact between the metal and semiconductor. When Φ_B_ is small or negative value, contact would be Ohmic. Φ_B_ between Ir and hydrogen-terminated single crystal diamond can be calculated by equation: Φ_B_ = χ + E_G_ − Φ_M_, where χ, E_G_ and Φ_M_ are electron affinities of diamond surface (−1.3~−1.4 eV)^37,38^, bandgap energy of diamond (5.5 eV) and work function of Ir (5.27 eV), respectively. According to this equation, Φ_B_ would be around −1.1 eV. Therefore, ohmic contact between Ir and diamond could be formed under large Ir work function. Further analysis of Φ_B_ between Ir film and hydrogen-terminated single crystal diamond will be reported in the future.

**Figure 6:**
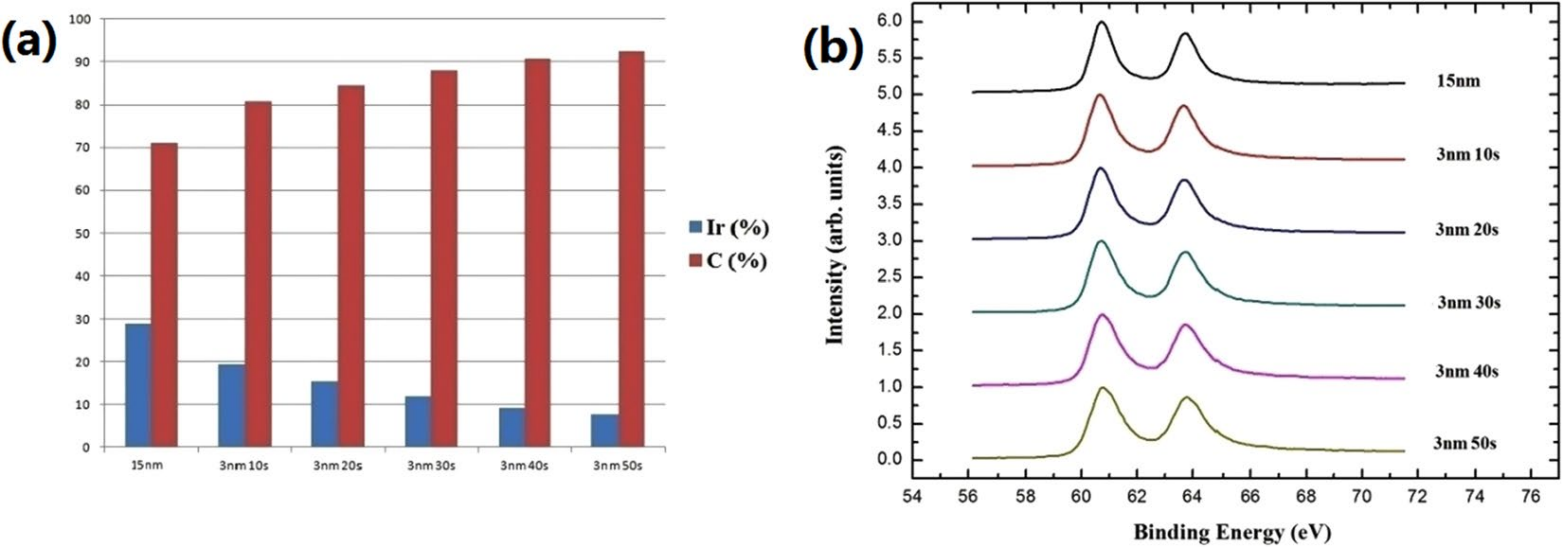
(**a**) Ir and C proportion with Ar etching; (**b**) XPS spectra of 15 nm Ir and 3 nm Ir with Ar etching 10 sec each time of sample D.

**Figure 7:**
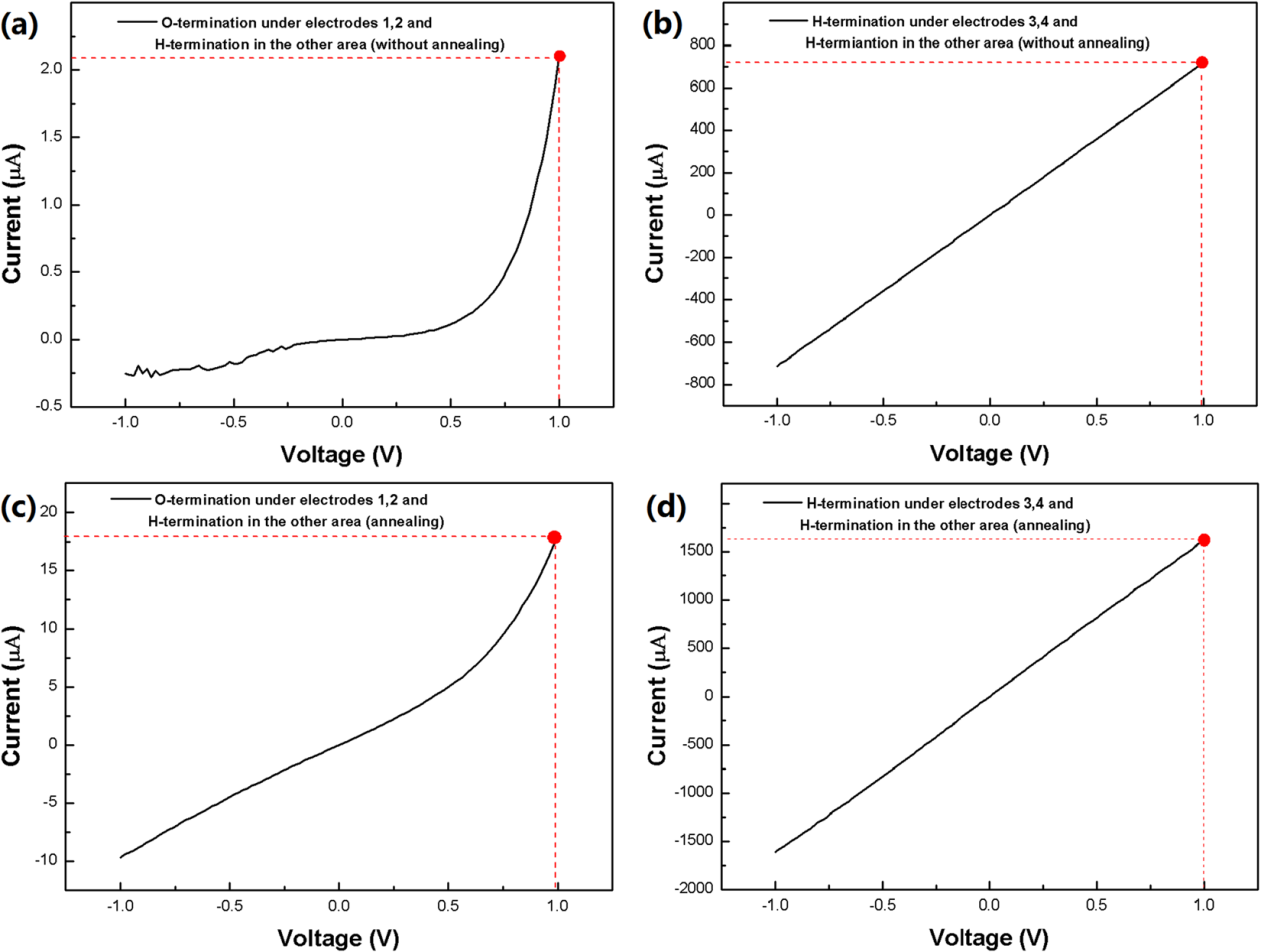
(**a**) I-V properties of electrodes 1, 2 before annealing; (**b**) I-V properties of electrodes 3, 4 before annealing; (**c**) I-V properties of electrodes 1, 2 after annealing; (**d**) I-V properties of electrodes 3, 4 after annealing of sample E.

Additional experiments were carried out to show if IrC formed and the H-termination existence even after the Ir deposition and annealing. IrC, after Ir deposition, could be detected by EDX spectroscopy elemental analysis showing no observed result in Fig. 5. In general, IrC formation temperature is more than 2000 °C, however, the annealing temperature was only 600 °C in this paper which is hard to form IrC between Ir and H-terminated diamond. In order to experimentally show that no IrC formed after Ir annealing at 600 °C, sample D was characterized by XPS technique. The investigation points were at the 3 nm Ir area and each of them was etched for 10 sec by Ar; each point was measured for 5 times. Figure 6(a) illustrates the proportional of Ir and C with Ar etching time of sample D, C% keep slightly increasing and Ir% gradually decreasing as etching time prolong. For instance, Ir changed from 20% to 7%, indicating that the Ir/diamond interface was detected by X-ray. 15 nm Ir XPS spectrum used as reference and sample D XPS spectra as shown in Fig. 6(b). After peak analysis technique, the XPS spectra of sample D shows just Ir 4f_7/2_, Ir 4f_5/2_ and Ir 5p_1/2_ peaks, indicating that only Ir exists at the Ir/diamond interface. Therefore, IrC was not formed after Ir deposition and annealing. In order to show that H-termination existence after all, sample E was prepared and measured. I-V properties of electrodes 1, 2 and 3, 4 with 1 V applied voltage on sample E before annealing shown in Fig. 7(a) and (b), the currents were about 2 µA and 900 µA, respectively, which electrode 3, 4 is 450 times higher. This result indicates that H-termination was still exist under electrodes 3, 4 after the Ir deposition. For comparison, I-V properties of electrodes 1, 2 and 3, 4 on sample E after annealing shown in Fig. 7(c) and (d), the currents were about 17 µA and 1.6 mA with 1 V applied voltage, respectively, which electrode 3, 4 is 100 times higher than that in electrode 1, 2. It could be concluded that H-termination existence under electrodes 3, 4 after the Ir annealing. Therefore, both experimentally results proved H-termination was still exist even after the Ir deposition and annealing. The current in Fig. 7(c) was larger than that in Fig. 7(a) with 1 V applied voltage. The current in Fig. 7(d) was larger than that in Fig. 7(b) with 1 V applied voltage. The currents increasing could be ascribed to the changing of defects in diamond surface during annealing process^39,40^.

## Conclusions

In summary, ohmic contact between Ir film and hydrogen-terminated single crystal diamond has been successfully realized and investigated under different annealing temperatures in Ar and hydrogen ambient. Considering brittle fracture of Ir, electrodes of Au/Ir (85/15 nm) have been fabricated. Based on TLM and EDX measurements, Ir film has potential to perform excellent ohmic contact with hydrogen-terminated single crystal diamond. The best ρ_b_ is 2.9 × 10^−6^ Ω cm^2^ annealing at 400 °C in Ar ambient. Two reasons could lead to the ohmic contact. One is no intermediate product formed during Ir film deposited on hydrogen-terminated single crystal diamond. The other is negative Φ_B_ between Ir film and hydrogen-terminated single crystal diamond.
